# An Individualized Right-to-Left Tunneling “Bail-Out” for Complex ICD Upgrade in a Pacemaker-Dependent Patient: A Case Report and Literature Review

**DOI:** 10.3390/jpm16060318

**Published:** 2026-06-14

**Authors:** Dimitrios A. Vrachatis, Konstantinos A. Papathanasiou, Sotiria G. Giotaki, Christos Piperis, Maria S. Kousta, Ioannis Anagnostopoulos, Christos Karavasilis, Gerasimos Deftereos, Georgios Giannopoulos, Sotirios Patsilinakos, Gerasimos Siasos, Spyridon Deftereos

**Affiliations:** 1 Electrophysiology and Pacing Department, Eugenideio Clinic, National and Kapodistrian University of Athens, 115 28 Athens, Greece; 2 Department of Biomedical Engineering, Medical School, National and Kapodistrian University of Athens, 115 28 Athens, Greece; 3 Third Department of Cardiology, Aristotle University of Thessaloniki, “Hippokration” General Hospital, 546 42 Thessaloniki, Greece; 4 Third Department of Cardiology, Medical School, National and Kapodistrian University of Athens, Sotiria Chest Disease Hospital, 115 28 Athens, Greece

**Keywords:** right-sided cardiac implantable electronic device, upgrade, pacemaker dependence, over the sternum tunneling

## Abstract

Inadequate vein access is a frequent obstacle during cardiac implantable electronic device (CIED) upgrade procedures; thus, bail-out strategies are employed. A 71-year-old male with dilated cardiomyopathy bearing a 7-year-old right-sided dual-chamber pacemaker was scheduled for upgrade to an implantable cardioverter defibrillator. The case presented two main challenges—first, pacemaker dependency, and second, an occluded right subclavian vein. In a shared decision-making approach, the decision was made to “abandon” the right-sided ventricular lead in situ, reposition the right-sided atrial lead by tunneling over the sternum into the left pectoral area, and implant a new left-sided defibrillator lead. During the 2-year follow-up our patient remained clinically stable and the CIED fully functional. Herein, beyond case presentation we also elaborate on individualized alternative treatment strategies for patients with venous access site occlusion in a literature review.

## 1. Introduction

Many patients undergo cardiac implantable electronic device (CIED) procedures in Europe [1]. While temporal trends vary worldwide, CIEDs are mainly inserted in elderly patients with multiple comorbidities [2,3]. Interventional complexity, operator experience and patient’s comorbidities are the main determinants of procedural complications and should be meticulously considered to prioritize patient’s safety [1].

CIED upgrade is frequently required to optimize heart failure management and prevent sudden cardiac death in patients with heart failure [1]. Long-standing CIEDs pose significant procedural challenges, including complex lead extraction and venous access site occlusion (VASO). VASO is commonly encountered during CIED reoperations and is often asymptomatic. Male sex and prolonged lead dwell time have been identified as independent predictors of VASO [4].

A variety of management strategies have been described. Common approaches are the contralateral lead implantation with subsequent tunneling to the original pocket, as well as the venous recanalization with or without lead extraction approach. Less frequently utilized strategies include the femoral or iliac vein approach with abdominal pocket creation, the internal jugular vein approach with supraclavicular tunneling, and the innominate vein approach [5,6,7].

Further, pacemaker dependency may also complicate management, necessitating thorough pre-procedural planning, particularly in patients with heart failure. This case report aims to elucidate the challenges and strategies involved in upgrading a pacemaker-dependent patient with a longstanding CIED to an implantable cardioverter defibrillator (ICD), specifically addressing the personalized considerations for lead management and device placement. Additionally, a literature review of bail out strategies in case of VASO is presented.

## 2. Case Presentation

A 71-year-old male with dilated cardiomyopathy and reduced left ventricular ejection fraction 25% was referred for ICD implantation (upgrade) for primary prevention in the context of elective generator replacement indication. Seven years prior to his current presentation, he received a right-sided dual-chamber pacing system due to high-grade atrioventricular block. Importantly, the patient was pacemaker-dependent.

During routine evaluation, echocardiography showed a dilated left ventricle (LVEDD 65 mm, LVESD 56 mm) with severely reduced systolic function (LVEF 25%) and moderate tricuspid regurgitation. Multiple episodes consistent with non-sustained ventricular tachycardia had been documented during pacemaker follow-up. Recent coronary angiography showed no obstructive coronary artery disease. The patient was in New York Heart Association class I and was receiving optimal medical therapy, including valsartan/sacubitril, dapagliflozin, carvedilol, and eplerenone. He had no family history of dilated cardiomyopathy, sudden cardiac death, or atrioventricular block before the age of 50. Genetic testing was not performed, and cardiac magnetic resonance imaging was unremarkable. In view of these findings, a shared decision was made to proceed with ICD upgrade for primary prevention of sudden cardiac death, in accordance with current guidelines [6].

The procedure was performed in June 2023. Given the longstanding right-sided CIED, the moderate tricuspid regurgitation and patient’s pacemaker dependency, our provisional plan was to retain the right-sided atrial lead, abandon the right-sided ventricular lead in situ and implant a right-sided double coil defibrillator lead. Continuous temporary pacing support was established via right femoral vein access. After appropriate pre-operative antibiotic prophylaxis and skin antisepsis, an ultrasound-guided right axillary vein puncture was performed. However, right subclavian vein was totally occluded preventing the advancement of wires. After venography confirmed that the left subclavian vein was patent, we considered changing our plan into implanting a left-sided single coil defibrillator lead into the right ventricle and transferring the functional right-sided atrial lead into the left pectoral area through a subcutaneous presternal tunnel. The patient was informed and provided his verbal consent.

Left axillary vein access was achieved via ultrasound guidance, and a single coil defibrillator lead was inserted into right ventricle and was set as a back-up pacemaker thereafter. After the construction of the CIED pocket into the left pectoral area, we returned to the right pectoral area where after opening the pocket and disconnecting the old CIED from the leads we confirmed that atrial lead measurements were intact. Subsequently, we embarked on the construction of the presternal subcutaneous tunnel for the atrial lead diversion. Since dedicated tunneling tools were not available, we had to follow a bail-out strategy consisting of gentle and successive blunt dissections with long hemostats. Starting from the left-sided incision near the atrial lead a subcutaneous path was steadily created towards the right-sided pocket avoiding any damage to intercostal muscles, ribs and sternum. To divert the atrial lead from right to left, a long (25 cm) splitable sheath introducer was utilized (Prelude Snap; Merit Medical Systems, Utah, USA). The introducer sheath was carefully advanced from left to right. The dilator was retracted and subsequently the atrial lead was gently retrogradely inserted (for a length ~0.5–1.0 cm) and stabilized with a hook created with a non-absorbable, multifilament silk 1.0 suture (external to the sheath). Afterwards the “system” was carefully pulled through the presternal tunnel into the left pectoral pocket and the atrial lead was released (the hook was cut with a scalpel blade no. 11). A critical step was to ensure that atrial lead was loose enough to prevent future damage during arm movements by the patient, while no adaptors were required for lead extension. Atrial lead measurements were repeated after the completion of the diversion and standard steps were followed including atrial lead anchoring to the left pectoral muscle. At the end of the whole procedure, we implicated a cap-and-bury approach for the abandoned right-sided ventricular lead to prevent migration and interference.

**Figure 1:**
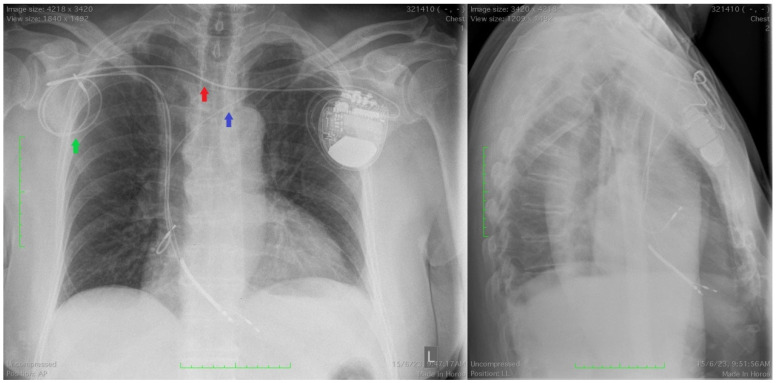
Legend: Post-procedural chest X-ray 24 h after implantation showing the tunneled presternal atrial lead (red arrow), the abandoned right-sided ventricular lead (green arrow) and the left--sided defibrillator lead (blue arrow).

**Figure 2:**
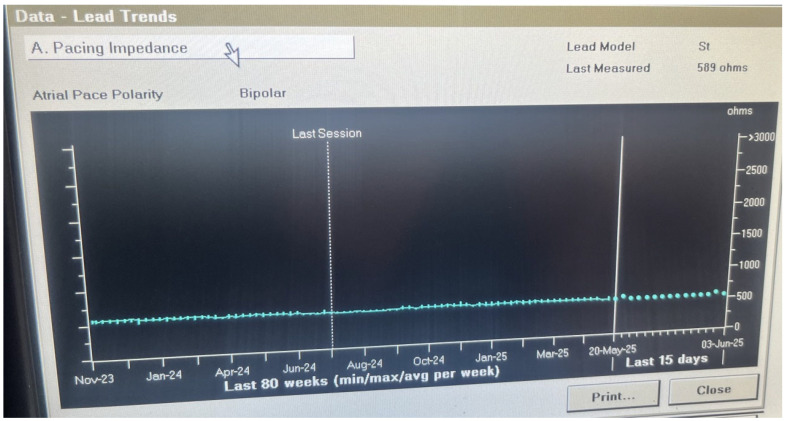
Legend: Stable atrial lead pacing impedance trend over 2 years of follow up.

**Figure 3:**
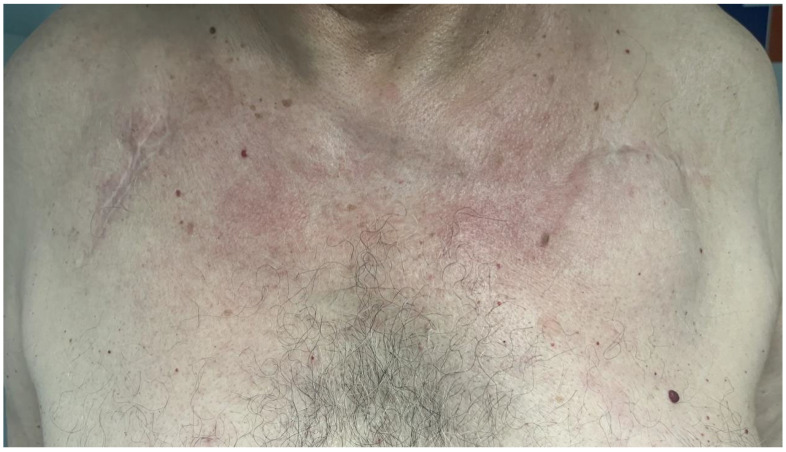
Legend: Uncomplicated incision healing in both pectoral areas.

The patient had an uncomplicated course and was discharged the following day. A post-procedural chest X-ray 24 h after implantation is shown in Figure 1. In-office reassessments were scheduled for one week and one month after the procedure. Having completed two years after the procedure, the patient remains clinically stable under a 6-month follow-up schedule. The lead measurements on June 2025 were as follows: P wave amplitude 1.6 mV, R wave amplitude 2.1 mV, atrial pacing impedance 589 ohms, ventricular pacing impedance 342 ohms and ventricular defibrillation impedance 70 ohms. Atrial pacing impedance trend is shown in Figure 2. Figure 3 depicts the current appearance of the pectoral incisions.

## 3. Discussion

### 3.1. Venous Access Site Occlusion in CIED Patients: Epidemiology and Pathophysiology

The convergence of an aging population and the escalating prevalence of heart failure—the most frequent morbidity among the elderly—has established CIED upgrades as a fundamental component of contemporary clinical management. Consequently, a growing cohort of patients requires complex procedures for lead revision, upgrade to ICD, or transition to cardiac resynchronization therapy (CRT).

Venous abnormalities, ranging from stenosis and thrombosis to total occlusion, are frequently encountered during these re-interventions [8]. These lesions are traditionally categorized by the degree of luminal narrowing, with mild stenosis affecting up to 40% of patients, while severe or total VASO is observed in 3–9% [8,9,10]. Recent data, such as the cohort study by Czajkowski et al., have introduced the concept of multi-level lead-related venous stenosis/occlusion as a more comprehensive index of global venous obstruction compared to single-point narrowing [11].

Clinically, lead-related venous obstruction remains predominantly asymptomatic, with the notable exception of acute axillary vein thrombosis [12]. However, its significance becomes paramount during device upgrades where venous access is critical. Established risk factors include total lead burden, the presence of coronary sinus leads, and abandoned leads, with prior ICD upgrades showing a higher incidence of occlusion [8,10]. Patient-specific factors, particularly male gender and the age at primary implantation, also significantly correlate with VASO [8,10,12].

Pathophysiology of these lesions is multifactorial and time-dependent. While acute thrombosis often occurs adjacent to the implantation site without pre-existing stenosis, late-onset stenosis (developing beyond the first year) typically results from the organization of a prior thrombus. Key mechanisms include (a) vascular ligation—cephalic vein ligation during a cut-down approach may facilitate thrombus extension into the central circulation, (b) hemodynamic stasis—slow flow within collateral circuits induces a prothrombotic state and (c) endothelial trauma—lead-induced mechanical trauma triggers a chronic inflammatory response within the vessel wall culminating in extensive scarring and fibrotic remodeling [8].

### 3.2. Lead Extraction Versus Abandonment Strategies

As previously stated VASO represents a frequent and clinically relevant challenge among patients undergoing CIED upgrade [13]. In this setting, the operator is often required to choose between transvenous lead extraction (TLE) and lead abandonment with alternative strategies for new lead implantation.

Each approach carries distinct advantages and challenges; hence, decision-making should be individualized. In the presence of device-related infection or endocarditis, TLE is clearly indicated. In addition, younger patients with long life expectancy, multiple dysfunctional leads, or leads requiring replacement are generally considered suitable candidates for extraction [8]. TLE allows preservation of the contralateral venous system and maintenance of magnetic resonance imaging (MRI) compatibility [8]. However, it requires surgical backup, is often performed under general anesthesia, and is associated with potentially serious complications, including cardiac tamponade, vascular injury, and procedural mortality [8].

Conversely, lead abandonment may be preferred in older or frail patients, in those with long lead dwell time, low overall lead burden, absence of infection, or increased procedural risk [8]. In such cases, the risks associated with TLE may outweigh its potential benefits. Nevertheless, abandonment may compromise future vascular access (especially among oncology patients and those requiring dialysis therapy), limit MRI compatibility, and complicate subsequent device management [14]. Furthermore, lead abandonment is associated with increased risk of CIED-related infection, venous obstruction (including progression to bilateral occlusion or superior vena cava syndrome) and tricuspid valve dysfunction [8]. All these potential complications should be carefully considered and discussed with the patient in a shared decision-making approach [14].

When lead abandonment is selected, several alternative strategies for lead implantation have been described. These include contralateral venous access with subcutaneous tunneling, supraclavicular or internal jugular approaches, as well as femoral access or venous recanalization techniques [8]. Among these, contralateral implantation with tunneling to the original pocket represents a widely adopted and relatively less invasive option [15,16,17].

Subcutaneous tunneling may serve as an effective bail-out strategy when conventional approaches are not feasible. By enabling the transfer of functional leads to a patent venous system, it avoids the risks associated with complex extraction procedures, especially in pacemaker-dependent or high-risk patients [8,14,17]. One practical consideration is to ensure CIED system compatibility and availability of adaptors or extenders in case of old dwelling leads.

It should be emphasized that venous recanalization represents the established strategy since it preserves contralateral venous access [8,14]. Venoplasty is typically performed using guidewire crossing techniques, often employing principles analogous to chronic total occlusion interventions. Following successful wire passage, balloon venoplasty can restore luminal patency and allow lead implantation. Pre-procedural venoplasty is highly effective (success rate > 90%) and safe, especially when employed in laboratories with interventional expertise accompanied with the assistance of an interventional cardiologist [18]. Worley et al. reported that venoplasty was successful in 371/373 patients presented with VASO without major complications or damage to the previously implanted leads. Hydrophilic wires were effective in crossing the sites of occlusion in most cases (>85%) and conventional noncompliant balloons were highly successful and safe [19]. In a small retrospective study Ji et al. compared an ICD recipient presented with VASO that underwent venoplasty versus patients that did not. They found that venoplasty was successful in all cases employed, while 1 out of 2 patients that did not undergo venoplasty required contralateral side implantation [19].

In selected cases, recanalization may be combined with TLE or performed using advanced techniques such as “wire externalization” or “inside-out” access [13,20]. Recent data form Swiss TLE registry suggest that TLE is highly successful (>95%) during CIED upgrade procedures and the complications remain low and are mainly related to the implantation stages and not the extraction stages [21]. Of note, Stefańczyk et al. reported that CIED upgrade can be completed with a 100% procedural success and zero major complications by mechanical means only (no laser or electrosurgical dissection sheaths were utilized) provided that the operators are experienced and venoplasty is available [22]. When initial extraction attempts based on mechanical sheaths and traction are ineffective, TLE can be completed with highly effective techniques such as the femoral approach with a dedicated snare [21], the internal transjugular approach [23] or the use of dedicated tools such as bidirectional rotational sheaths [24] or laser-assisted extraction tools [24,25,26,27].

However, TLE procedures are technically demanding, time-consuming, and require specific expertise (cardiothoracic surgical back-up, interventional radiology or cardiology support and occasionally general anesthesia) and specialized equipment, which may limit their applicability in routine clinical practice [13]. Of note, long-term mortality remains high after TLE, especially among elderly patients (>75 years old), CIED infection indication, heart failure, chronic kidney disease and long lead dwell time [28].

#### Pre-Sternal Tunnel and Contralateral Venous Access

Our case highlights several critical considerations in contemporary CIED management, in particular lead extraction, pacemaker dependence, and defibrillator implantation site efficiency. The decision to abandon the old right-sided ventricular pacing lead while implanting a new left-sided ICD was a complex one. Table 1 summarizes the considerations in favor and against the construction of a presternal tunnel in our patient.

This decision reflects a pragmatic approach given lead’s dwell time and the absence of an absolute indication for extraction such as infection or malfunction. The primary driver for intervention in our case was the need for a CIED with defibrillation capability, which the old device lacked. In addition, the patient’s pacemaker dependence further complicated this decision. The insertion of temporary pacing wires itself carries risks, such as pneumothorax, vascular complications and infection. Any complication during lead removal in a pacemaker-dependent patient with heart failure could lead to adverse events, as the patient’s impaired systolic function renders them more vulnerable to acute stressors. Recently published studies indicate that TLE in patients with abandoned leads is associated with increased procedural complications often necessitating advanced tools or surgery [7]. Nevertheless, a sterile lead abandonment strategy is not associated with reduced 10-year survival compared to an initial lead extraction strategy [29].

A left-sided implantation should be prioritized due to risk of increased all-cause mortality [30] with right-sided ICDs and sub-optimal shock vector [31,32]. However, comorbidities such as end-stage kidney disease requiring renal replacement therapy or chemotherapy-treated patients should be considered case by case. In case of a right-sided ICD defibrillation threshold testing [31,32], wavefront optimization should be considered [31,32].

The tunneling technique was initially described in 1983 by Belott during an attempt to treat pacemaker syndrome among VVI-paced patients [33], and in 2006 Fox et al. proposed that a tunneled contralateral left ventricular lead placement could be useful during CIED upgrade procedures [14]. Two previously published cohorts including 45 patients (47 tunneled leads) indicated that ≥ 95% (45/47) of the presternally tunneled leads remain stable after 2–3 years of follow-up [17,34]. Table 2 describes additional cases utilizing this approach in different clinical settings.

Alternative bail out strategies have been described, including ispilateral puncture proximal to the chronic subclavian obstruction site [35] or contralateral innominate vein puncture [36]. Table 3 summarizes previously published cases employing these medial to VASO or supraclavicular approaches.

Femoral vein access has also been suggested as an alternative approach in case of occluded upper body central veins. Of note, the intubating catheter is able to straighten out curves leading to the coronary sinus and it consequently provides excellent catheter support by forging a linear path to the coronary sinus [37].

Increasing evidence supports the utilization of recanalization techniques and subclavian venoplasty [13,20,38]. In these techniques, a guidewire is advanced through the stenosis either in an antegrade or retrograde fashion [8,38]. Once the wire crosses the stenotic lesion, dilation or venoplasty is performed [20]. Specialized expertise, dedicated equipment and vascular surgery backup are essential due to the risk of perforation during balloon inflation or lead advancement following venoplasty [20]. Nevertheless, when tunneling is unfeasible or when preserving the existing venous access is critical to prevent compromising collateral drainage, venoplasty is considered a viable alternative [20,38].

Lastly, subcutaneous or extravascular lead implantations remain a valuable approach provided that device interaction issues are addressed with careful programming of both devices [13,39]. According to the PRAETORIAN extended follow-up trial, subcutaneous ICDs show no significant difference in overall device-related complications compared to transvenous ICDs after a median of approximately 7 years [40]. However, transvenous ICDs are associated with a higher risk of lead-related complications. Consequently, the subcutaneous ICD represents a valid alternative for patients without a pacing indication [40]. Moreover, the novel modular cardiac rhythm management system combines subcutaneous ICD with a leadless pacemaker capable of delivering anti-tachycardia pacing and a recent trial suggested that this approach is both safe and effective after a median follow-up of two years [41].

Data from a recent meta-analysis support the utilization of extravascular ICDs which demonstrate a high feasibility rate (97%), high success rate in terminating ventricular arrhythmias at implant (99%) and a low incidence of periprocedural complications (4%) [42]. Of note, the extravascular ICD Pivotal study showed that after 3 years of follow-up ATP efficacy in terminating ventricular tachycardia episodes is 77%, while shock therapy was successful in all cases. Inappropriate therapies were not negligible approaching 18% and were mainly attributed to P wave oversensing [43].

As a conclusion, it must be underscored that decision-making process in CIED management is intricate. The importance of individualized patient care through a thorough risk–benefit assessment should be emphasized. Lead extraction and recanalization remain the preferred strategy for patients with VASO, especially when the patient is young, without comorbidities or in case of CIED-related infection. Pre-sternal tunneling should only be considered in frail patients featuring long lead dwell time and increased risk of major cardiovascular complications, especially in hospital settings without cardiothoracic surgical back-up. Our bail-out presternal tunneling approach was primarily based on unavailable extraction tools and surgical backup. This strategy is by no means equivalent to standard extraction-based management and should be only adopted under specific organizational constraints.

**Table 1:** Factors considered during lead management.

Favoring Tunnel Construction	Against Tunnel Construction
***Patient-related factors***	***Patient-related factors***
Severe left ventricular dysfunction	Non-MRI conditionality
Patient’s age	
***Procedural and CIED-related factors***	***Procedural and CIED-related factors***
Non-ICD lead for contralateral tunneling	Skin erosion
Low total number of leads (≤4)	Lead to lead abrasion or interaction lead malfunction
Complete vein occlusion	Endocarditis risk
Optimized vector of the defibrillation shock with a left-sided system	Lead-related thrombus formation
No cardiothoracic surgical back-up	CIED-related tricuspid regurgitation
Risk of major complications (hemothorax, tricuspid valve damage, tamponade)	Challenging lead extraction (in case of an absolute indication for extraction)
Potential need for removal of all functional leads instead of only a redundant atrial lead in case of inter-lead fibrosis	Risk of bilateral subclavian vein obstruction

CIED: cardiac implantable electronic device, ICD: implantable cardiac defibrillator, MRI: magnetic resonance imaging.

**Table 2:** Case reports and cohorts evaluating tunneling of leads through a contralateral subclavian vein approach.

Author, Year	Access	Implantation Indication	Demographics	Comorbidities	CIED Type	Lead Type	Periprocedural Complication	Follow-Up
Belott [33], 983	Contralateral SVP (n = 2)	PPM syndrome AV dyssynchrony	N = 2	HF (n = 2)	PPM (n = 2)	Atrial (n = 2)	Not reported	Not reported
Fox [14], 2006	Contralateral SVP (n = 2)	Upgrade ICD to CRT	N = 2 58 y, male 38 y, male	HFrEF (n = 2) CAD (n = 1)	CRT (n = 2)	LV lead (n = 2)	No	6–12 months consistent biventricular pacing and clinical improvement
Kim [44], 2010	Contralateral SVP (n = 1)	Aborted sudden cardiac death	N = 1 56 y, male	HTN, DM, CAD	ICD (n = 1)	dual coil ICD lead (n = 1)	No	24 months stable lead parameters no therapies delivered
Luthje [17], 2011	Contralateral SVP (n = 18)	Lead dysfunction (n = 8) CIED upgrade (n = 6) device relocation (n = 4)	N = 18 66 ± 14 y 12 males	Ischemic HF (n = 9) DCM (n = 4) Valvular HD (n = 1) Congenital HD (n = 2) Other HD (n = 2)	PPM (n = 1) ICD (n = 9) CRT (n = 8)	ICD lead (n = 7) LV lead (n = 5) RV lead (n = 4) Atrial lead (n = 4)	No	3–162 months 3 patients with mild wound complications or discomfort
Goli [45], 2013	Contralateral SVP (n = 1)	Non-ischemic HEART FAILURErEF LVEF 17% QRS 189 ms	64 y, male	DCM severe MR	CRT (n = 1)	LV lead (n = 1)	No	6 months decreased BNP levels and clinical improvement
Sadarmin [15], 2015	Contralateral SVP (n = 2)	Upgrade ICD to CRT (n = 2)	N = 2 65 y, male 78 y, male	CAD (n = 1) COPD (n = 1) HFrEF (n = 2) LBBB (n = 1) QRS > 130 (n = 2)	CRT (n = 2)	LV lead (n = 2)	No	12 months clinical improvement LV leads with stable parameters
Antoun [16], 2024	Contralateral AVP (n = 1)	Pacemaker-induced cardiomyopathy (LVEF < 40%)	73 y, male	SSS CAD	CRT (n = 1)	LV lead (n = 1)	No	4 months improved LVEF 50% and clinical improvement
Mekary [34], 2025	Contralateral vein access	CIED upgrade (n = 18) Lead revision (n = 8) First implantation (n = 1)	N = 27 69 ± 18 y 20 males	Non-ischemic HFrEF (n = 16) CAD (n = 12) AF (n = 11) CKD (n = 5) LVEF 29 ± 11%	CRT (n = 20) ICD (n = 5) PPM (n = 2)	LV leads (n = 20) ICD leads (n = 5) RV leads (n = 2)	Small hematoma (n = 2) (no intervention required)	24 ± 16 months One tunneled ICD lead had low shock impedance 3 years after implantation (no revision required)

AF: atrial fibrillation, AV: atrioventricular, AVP: axillary vein puncture, BNP: beta natriuretic peptide, CAD: coronary artery disease, CKD: chronic kidney disease, DCM: dilated cardiomyopathy, CIED: cardiac implantable electronic device, CRT: cardiac resynchronization therapy, DM: diabetes mellitus, ICD: implantable cardiac defibrillator, HD: heart disease, HTN: hypertension, HF: heart failure, HFrEF: heart failure with reduced ejection fraction, LBBB: left bundle branch block, LV: left ventricular, LVEF: left ventricular ejection fraction, MR: mitral regurgitation, ms: millisecond, N: number of patients, PPM: permanent pacemaker, RV: right ventricle, SSS: sick sinus syndrome, SVP: subclavian vein puncture, y: years.

**Table 3:** Case reports and cohorts evaluating medial to obstruction (supraclavicular) approaches.

Author, Year	Access	Implantation Indication	Demographics	Comorbidities	CIED Type	Lead Type	Periprocedural Complication	Follow-Up
Ovadia [46], 2000	Innominate vein (n = 4) Jugular vein (n = 1)	Complete AVB (n = 4) Secondary prevention (n = 1)	N = 5 4 children 6 y (3–9) 4 males 1 adult 70 y, male	Congenital HD (n = 4) Ischemic HFrEF (n = 1)	PPM (n = 4) ICD (n = 1)	RV leads (n = 4) Atrial leads (n = 4) ICD (n = 1)	Lead dislodgment (n = 1) phrenic capture (transient) (n = 1) hematocrit drop (n = 2) no transfusion required	7–28 months No major complications
Pires [47], 2005	Jugular vein (n = 10)	CAS obstruction (N = 5) CIED infection (n = 2) Preexisting right side CIED (n = 3)	N = 10 66 ± 15 y 6 males	HFrEF (n = 15) CAD (n = 5) LBBB (n = 8) IVCD (n = 2)	CRT (n = 10)	LV lead (n = 10)	Pocket hematoma (n = 1) (no intervention required)	12 months Increased LV pacing threshold (2.0 ± 0.9 V; *p* = 0.004)
Bosa-Ojeda [48], 2007	Jugular vein (n = 1)	Non-ischemic HF LVEF: 42% QRS: 180 ms	60 y male	Obesity	CRT (n = 1)	LV lead (n = 1)	No	12 months clinical improvement QRS: 150 ms no local symptoms
Aleksic [36], 2007	Innominate vein (n = 7)	Lead dysfunction (n = 4) Upgrade CRTD (n = 2) CIED infection (n = 1)	N = 7 72 ± 10 y 6 males	N/R	ICD (n = 5) CRT (n = 2)	N/R	Pneumothorax (n = 1)	16 ± 6 months No complications and complaints
Antonelli [35], 2010	Ipsilateral supraclavicular SVP (n = 4)	Pacemaker syndrome (n = 1) CRTD upgrade (n = 1) Lead dysfunction (n = 2) Secondary prevention (n = 1)	N = 4 65 ± 11 y 3 males	CAD (n = 4) HFrEF (n = 1)	ICD (n = 3) CRTD (n = 1)	Atrial lead (n = 2) LV lead (n = 1) ICD lead (n = 3)	No	24 months No complaints and stable lead parameters

AVB: atrioventricular block, CAD: coronary artery disease, CAS: cephalic-axillary-subclavian, CIED: cardiac implantable electronic device, CRTD: cardiac resynchronization therapy and defibrillator, ICD: implantable cardiac defibrillator, HD: heart disease, HF: heart failure, HFrEF: heart failure with reduced ejection fraction, LBBB: left bundle branch block, LV: left ventricular, LVEF: left ventricular ejection fraction, ms: millisecond, N: number of patients, N/R: not reported, IVCD: intraventricular conduction delay, PPM: permanent pacemaker, RV: right ventricle, SVP: subclavian vein puncture, y: years.

## Data Availability

Data available upon reasonable request.
